# Case Report: Intoxication in a Pig (*Sus Scrofa Domesticus*) After Transdermal Fentanyl Patch Ingestion

**DOI:** 10.3389/fvets.2020.611097

**Published:** 2020-11-24

**Authors:** Jerneja Sredenšek, Maša Bošnjak, Urša Lampreht Tratar, Tina Kosjek, Maja Cemazar, Mojca Kržan, Alenka Seliškar

**Affiliations:** ^1^Small Animal Clinic, Veterinary Faculty, University of Ljubljana, Ljubljana, Slovenia; ^2^Department of Experimental Oncology, Institute of Oncology Ljubljana, Ljubljana, Slovenia; ^3^Department of Environmental Sciences, Jožef Stefan Institute, Ljubljana, Slovenia; ^4^Jožef Stefan International Postgraduate School, Ljubljana, Slovenia; ^5^Faculty of Health Sciences, University of Primorska, Izola, Slovenia; ^6^Faculty of Medicine, Institute of Pharmacology and Experimental Toxicology, University of Ljubljana, Ljubljana, Slovenia

**Keywords:** intoxication, pig, naloxone-opioid overdose, pharmacokinetics, fentanyl transdermal patch

## Abstract

An experimental study on the effects of electroporation on pancreatic tissue was performed in pigs, and the fentanyl transdermal patch (FTP) was used postoperatively as part of multimodal pain management. Ingestion of an FTP, which resulted in fentanyl intoxication, was suspected 5 days after placement in one of the experimental pigs. The pig was first dysphoric, running in the stall, panting and vocalizing until it finally became depressed and it remained lying on the floor. Ingestion of an FTP was not observed but the fentanyl plasma concentration on the day of intoxication was 20.7 ng/ml, while at its peak after FTP administration it was only 0.492 ng/ml. The intoxication was successfully treated with a single intramuscular naloxone injection.

## Introduction

Fentanyl is a lipophylic μ-opioid receptor agonist. It is not approved for use in veterinary medicine, but it is used off-label for the treatment of acute pain following trauma or surgery and for chronic pain management in dogs and cats (1). Its use in pigs is limited (2–6). Accidental intoxication after ingestion of an FTP has been reported in a dog (7) and in non-human primates (8). This case report describes intoxication in a pig after suspected ingestion of an FTP and successful treatment with the opioid receptor antagonist naloxone.

## Case Presentation

A 32.1 kg female pig (*Sus scrofa domesticus*), a hybrid of Landrace and Large White, aged 10 weeks, was enrolled in an experimental study on the effects of electroporation and electrochemotherapy (9, 10) on pancreatic tissue (11). In compliance with the 3Rs principle (replacement, reduction, and refinement), blood samples for the pharmacokinetics of fentanyl and carprofen (12) were collected in addition to the electroporation procedure. The animal was procured from an authorized swine breeder 10 days before the procedure and housed in an individual indoor straw-bedded pen (1.25 × 4.40 m). The pen allowed visual and audible contact with other pigs. The pig was exposed to a natural light/dark cycle and kept at a room temperature of 20–23°C and a relative air humidity of 50–65%. It was fed twice daily with commercial feed and had unlimited access to tap water from nipple waterers.

The pig was fasted for 12 h prior to anesthesia, and water was offered *ad libitum*. It was sedated with ketamine (Bioketan, Vetoquinol, Lure Cedex; 10 mg/kg), midazolam (Midazolam Torrex; Torrex Chiesi Pharma GmbH; 0.5 mg/kg), and medetomidine (Domitor, Orion Corporation; 0.02 mg/kg), all of which were mixed in the same syringe and administered intramuscularly (IM). Next, a 20-gauge venous catheter was placed in left lateral auricular vein. Anesthesia was induced with isoflurane (Isoflurin, Vetpharma Animal Health S.L.) delivered in oxygen 5 L/min using a facemask. The pig was intubated endotracheally, connected to a circle breathing system and anesthesia was maintained with isoflurane (1.5–2%, vaporizer setting) delivered in 100% oxygen. Arterial blood pressure (Doppler flow monitor, Model 811; Parks Medical Electronics), end-tidal carbon dioxide concentration, arterial oxygen saturation, esophageal temperature, and electrocardiography were monitored (BLT M9000 VET; Guangdong Biolight Meditech). Enrofloxacin (Enroxil, Krka; 7.5 mg/kg) was injected IM after intubation. Normal saline (0.9% NaCl, B Braun, Melsungen; 10 ml/kg/h) and Voluven 6% (Fresenius Kabi; 4 ml/kg/h) were infused during anesthesia. For the electroporation procedure, the pig was placed in dorsal recumbency on an electrically heated surgery table and in sternal recumbency during pre- and post-procedure CT scans. Ketamine 0.3–0.4 mg/kg intravenously (IV) was given three times during anesthesia when the heart rate or blood pressure increased by 20% or more due to the procedure. Total anesthesia time was 4 h.

**Figure 1:**
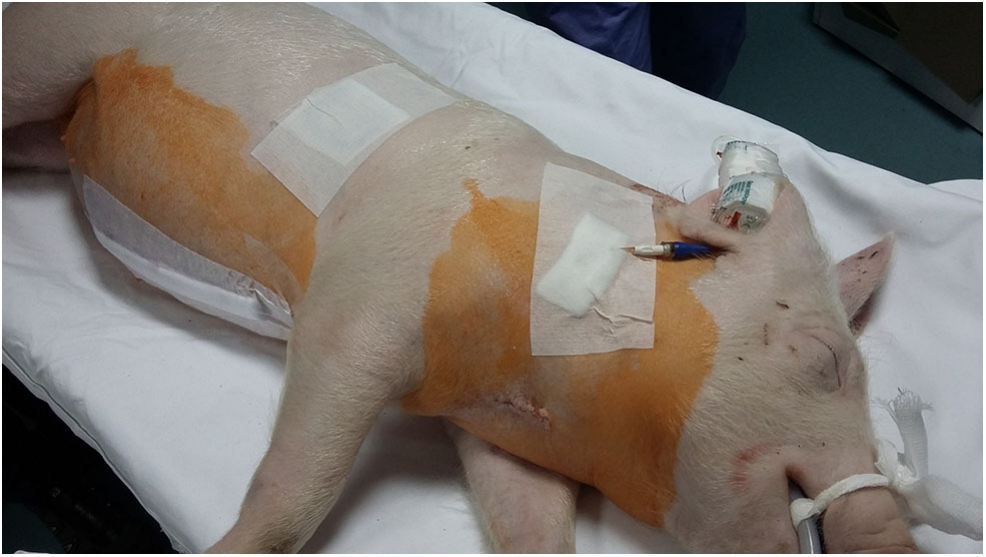
The pig at the end of the procedure. The FTP 75 μg/h was covered with a surgical adhesive dressing. It was planned to be left *in situ* until the end of experiment at Day 8, but it had become dislodged and was presumably ingested at the most 4 h before the signs of intoxication were observed on Day 5. FTP, fentanyl transdermal patch.

Before the electroporation procedure, a 16-gauge permanent central venous catheter (Intramedicut 2, Sherwood Medical) was surgically inserted in the right external jugular vein, channeled subcutaneously and led externally to the back of the neck where it was fixed and covered with a self-adhesive plaster. The catheter was flushed with normal saline and locked with trisodium citrate (Citra-Lock 30%, Dirinco B.V.) diluted with normal saline to a 3% solution. After placement of the central venous catheter, carprofen (Rycarfa, Krka; 4 mg/kg) was administered IV, and a 75 μg/h FTP (Durogesic, Janssen MD) was placed on the right side of the thorax caudally to the scapula. The skin was clipped, cleaned with tap water, and dried prior to patch attachment. The patch was covered with a surgical adhesive dressing (Figure 1). The plan was to leave it *in situ* until the end of the experiment at Day 8 after the procedure. A CT scan was performed before and after the electroporation procedure. The pancreas was surgically exposed through a median laparotomy, and ultrasound guided electroporation was performed. Before the end of the surgery, the wound edges were infiltrated with levobupivacaine (Chirocaine 0.75%, AbbVie; 0.6 mg/kg). Morphine (Morphini Chl., Alkaloid; 0.2 mg/kg) was injected IM at the end of the procedure and repeated once 4 h later, when the pig was already fully recovered. Carprofen (Rycarfa Flavour 100 and 20 mg/kg tablets with meat aroma, Krka; 4 mg/kg) was given orally once per day from Day 2 until Day 7 after the procedure. The tablets were crushed, mixed with plum jam, and offered on a spoon.

**Figure 2:**
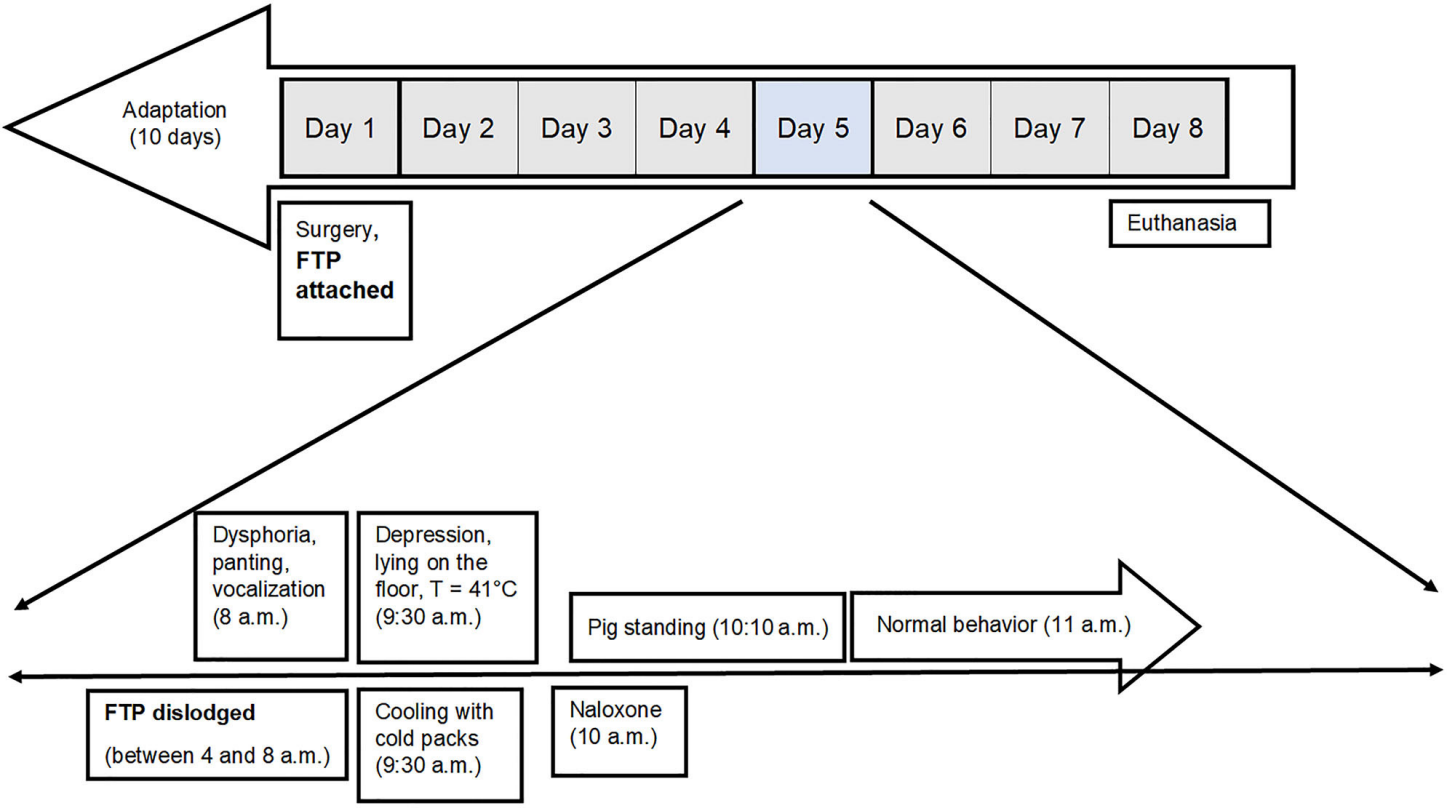
Timeline of events that happened on the day of intoxication of the pig with FTP 75 μg/h. Ingestion of the FTP could not be confirmed, however, the intoxication due to ingestion of FTP was suspected, and the pig was treated with naloxone. FTP, fentanyl transdermal patch.

The pig was under continuous video surveillance. The central venous catheter and FTP were checked three times a day. Over the night from Day 3 to 4 after the procedure, the catheter was dislodged, and until the end of experiment, the pig was fixed with a restraining snare for blood sampling. On Day 5 at 8 a.m., the animal keeper noticed that the pig was behaving strangely. It was dysphoric, running in the stall, panting, and vocalizing. Abrasions of the ventral aspect of the carpus and metacarpus of both front legs, caused by vigorous attempts to climb the fence, were observed. Within 90 min the pig became depressed until it remained lying on the floor, unable to rise upon vocal stimulation and gentle poking with the hand. At that time, the rectal temperature was 41°C, and cooling with cold packs was initiated. The pen was searched for the FTP, but it was not found. The video surveillance records were examined, and it was established that the FTP had become dislodged after 4 a.m. Ingestion of the FTP could not be confirmed, however, intoxication due to ingestion of the FTP was suspected, and a single dose of naloxone (Nexodal, Orpha-Devel Handels, und Vertriebs GmbH) 0.012 mg/kg was injected IM at 10 a.m. Approximately 10 min later, the pig rose to its feet, and within an hour normal behavior was restored (Figure 2). Until the end of the experiment, no other deviations from physiological pig behavior, appetite, urine voiding, and defecation were observed.

On Day 8, the pig was re-anesthetized using the same protocol as that used previously, and a CT scan of the abdomen was performed. Afterwards, it was euthanized with T61 euthanasia solution (Intervet, Boxmeer; 3 ml/10 kg) IV, and the pancreas was explanted for the purpose of the primary study.

During the study, blood was collected for the pharmacokinetics of fentanyl and carprofen. Blood samples were collected into heparinized tubes (Vacuette, Greiner Bio-One GmbH), the first one 30 s before FTP administration (time point 0) and at 30 and 60 min and 2, 4, 6, 8, 12, 24, 36, 48, 60, 72, 96, 120, 144, and 168 h thereafter. At each sampling, 3 ml of catheter lock solution and the first 2 ml of blood were discarded, and the blood sample was collected. The samples were centrifuged within 10 min after collection at 1,500 rpm and 20°C for 10 min. Afterwards, they were separated into aliquots and stored at −20°C until analysis. After each blood sampling, the catheter was flushed with normal saline and locked again. On Day 5, when intoxication by the FTP was suspected, the blood sample was collected at 9:30 a.m. (120 h after FTP administration and at most 5.5 h after its dislodgment).

Plasma samples (200 μL) were denaturated and prepared by solid phase extraction using Phenomenex Strata XC cartridges. The analyte was eluted off the sorbent using 5% triethylamine in acetone and 5% triethylamine in methanol. The extract was then reconstituted in a 200 μL acetonitrile/water (1/9) mixture with 0.1% formic acid. A Shimadzu Nexera X2 ultra-high performance liquid chromatograph (LC) coupled to a Sciex QTRAP 4500 hybrid quadrupole-linear ion trap mass spectrometry (MS) analyzer was employed for the analysis. LC separation was achieved by gradient elution on a Supelco Ascentis Express C18 column (50 × 2.1 mm, 2 μm). The mobile phases were 0.1% formic acid and acetonitrile. MS detection was performed after positive electrospray ionization in multiple reaction monitoring acquisition mode, using fentanyl transition 337 > 188 for its quantitation. The limit of quantification (LOQ) was 0.003 ng/mL and the limit of detection (LOD) was 0.001 ng/mL.

**Figure 3:**
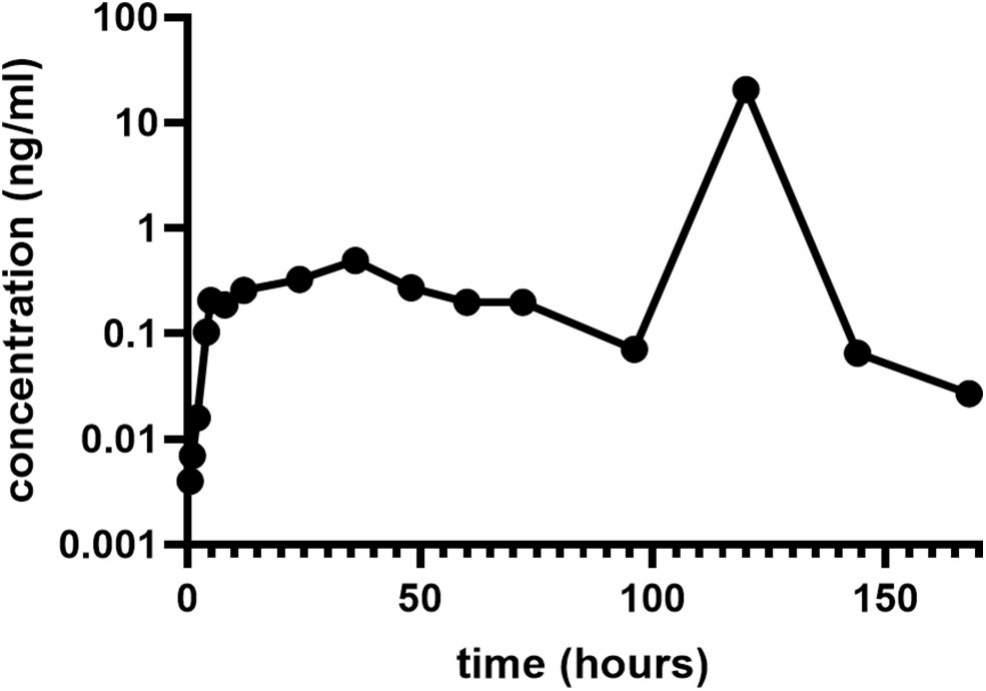
Concentration-vs.-time profile of fentanyl absorbed from a 75 μg/h FTP. On Day 5 (120 h) there was a sharp rise in plasma fentanyl concentration due to ingestion of the FTP. FTP, fentanyl transdermal patch.

Fentanyl was slowly absorbed through the skin, reaching a maximal plasma concentration (C_max_) of 0.492 ng/ml at 36 h (T_max_, time at which C_max_ is observed) post administration (Figure 3). Plasma steady state concentration measured 48, 60, and 72 h after administration of the patch varied between 0.199 and 0.273 ng/ml. On Day 5 (120 h) there was a sharp rise in plasma fentanyl concentration (20.7 ng/ml) as a consequence of ingestion of the FTP. This was followed by a rapid fall to levels below steady state [0.065 ng/ml on Day 6 (144 h) and 0.027 ng/ml on Day 7 (168 h)].

## Discussion

Fentanyl is an opioid analgesic whose action is based on the stimulation of μ-opioid receptors. Durogesic is a fentanyl matrix transdermal system with a drug-in-adhesive formulation designed to release fentanyl continuously after application to intact skin (13). It is approved for use in people, but it has also been used off-label in different animal species (2–6, 14, 15). There are only a few whole animal studies investigating the pharmacological properties of FTP in pigs, reporting wide variations in plasma concentration in individual animals (2–6).

The pig in this case report was included in a study where postoperative pain was expected to be severe because of a midline laparotomy and possible pancreatic inflammation due to an electroporation procedure (11). The fentanyl transdermal patch was chosen as a part of a multimodal analgesia protocol sufficient to permit normal behaviors and feeding and also to avoid stress due to frequent IM injections of morphine postoperatively.

The C_max_ of 0.492 ng/ml fentanyl was in the range (0.3–0.6 ng/ml) which other investigators (2) associated with adequate analgesia regarding post-operative pain score, food consumption, activity, and rate of movement in pigs that underwent thoracotomy and were administered a 50 μg/h FTP. Another study (3) determined the C_max_ of fentanyl (1.28 ± 0.84 ng/ml) at 6 h after the application of a 75 μg/h FTP to the interscapular area in 27.8 ± 2.2 kg pigs. Thirty-six hours after removal of the first FTP, a second FTP was applied to the same pigs which underwent ovariectomy. A much lower C_max_ of fentanyl (0.45 ± 0.08 ng/ml) was determined at a T_max_ of 36 h. The behavior of pigs that underwent ovariectomy did not differ significantly from those not ovariectomized. No rescue analgesia was administered in any group; however, the authors recognized that the pain scoring system they employed (2, 5) may not have been a sufficiently sensitive indicator of pain in pigs. A pain scale for the assessment of pain was not used in our study; however, the pig behaved normally, interacted well with caretakers, showed interest in the environment and other pigs postoperatively, and did not pay extra attention to the wound site. Food intake did not decrease after the procedure and, as a matter of fact, the pig gained 6.3 kg in 7 days. This is significantly more weight gain than that observed by other investigators (4), where pigs (20 ± 4 kg) which were treated with epidural morphine (0.1 mg/kg) preoperatively and a 50 μg/h FTP applied behind the ear immediately after abdominal surgery gained 0.5 ± 0.2 kg during the subsequent two postoperative days. Considering that in our pig an opioid-sparing multimodal approach including carprofen was used, this might have contributed to the sufficient postoperative analgesia and good weight gain.

The FTP in this study was placed on the right side of the thorax caudally to the scapula, and the pig was not able to reach it with its snout (Figure 1). The pig was housed in an individual pen, excluding the possibility that other pigs could remove the patch. The day before intoxication, the plasma concentration of fentanyl was already low (0.071 ng/ml). A layer of FTP that is adhering to the skin contains a drug-in-adhesive reservoir, which contains fentanyl. The concentration gradient that exists between the patch and the skin drives drug release (13). In reservoir there is enough fentanyl to provide a sufficient concentration gradient for transdermal absorption over 3 days after application (16, 17). It is recommended to change the patch every 72 h if further analgesia is required (13). The FTP in our pig was planned to be left *in situ* until the end of experiment due to the requirements of primary pharmacokinetic study.

A sufficient amount of fentanyl to cause death in people was present in a 100 μg/h FTP (28–84.4% of the original contents) after 3 days of continuous use (18). Likewise, in dogs the FTP contained an average of 83% of the original amount after 72 h (19). FTPs applied to the skin are not clinically useful after 3 days, but there is still a significant residual amount of fentanyl present in Durogesic patch to cause an intoxication if chewed or ingested (8, 20, 21). If tissue is lacking stratum corneum (e.g., mucosa), this increases fentanyl absorption for more than 30-fold (22). Systemic absorption is also increased by chewing an FTP because the contact time with the buccal and sublingual mucous membrane is prolonged and because chewing liberates the drug from the reservoir (20). After 72 h FTPs should be removed and folded so that the adhesive side of the patch adheres to itself (13). There are different suggestions for disposing FTPs. Some authors suggest returning the patch to the pharmacy or flushing it down the toilet (16), while others suggest incineration or cutting it to pieces before discharging it (23).

In case reports of fentanyl overdose after FTP ingestion in a dog (7) and two non-human primates (8), fentanyl plasma concentrations were 1.7 ng/ml, and 8.29 and 14.8 ng/ml, respectively. A 29 kg dog was suspected of ingesting the contents of the reservoir of a 100 μg/h FTP (reservoir-type patch), and fentanyl was absorbed across the oral mucosa or gastrointestinal tract. About 38 h after the placement of the FTP and ~7 h after the last time the FTP was observed to be intact, the dog presented with extreme sedation and bradycardia, but it recovered with symptomatic therapy only (7). In two non-human primates weighing 3.5 kg, a 25 μg/h FTP caused severe bradypnea, followed by apnea. This occurred when they were re-anesthetized with ketamine 20 and 23 h after the placement of the FTP, respectively. Both animals were intubated and ventilated, but they died despite intensive supportive therapy and several attempts to reverse fentanyl with naloxone. At necropsy, the FTP was discovered in each animal's cheek pouch (8). The risk or severity of adverse effects can be increased when fentanyl is combined with ketamine (24), which probably contributed to the unsuccessful resuscitation.

It is possible that the pig removed the FTP while rubbing its body against the wall of the pen, since the central venous catheter was placed on the same side of body as the FTP, and mild inflammation due to subcutaneous tunneling of the catheter might have caused itching. Skin reactions and pruritus at the site of FTP application have been reported in humans and animals (1, 13). Pruritus is a well-established side effect of fentanyl, although it occurs more often after intrathecal or epidural than transdermal application (13). However, the skin under and around the FTP was not visibly irritated in our case. We inspected the pen and the pig's mouth but failed to find the FTP. Based on the clinical signs, response to naloxone treatment and high fentanyl plasma concentration (20.7 ng/ml) on the day of intoxication, we assume the FTP was ingested.

In our case, we were not able to find the patch, but given the clinical signs (first dysphoria and hyperthermia and then central nervous system depression), we suspected fentanyl intoxication due to FTP ingestion. Fentanyl plasma concentration at most 5.5 h after dislodgment of the FTP was much higher than in both the intoxicated dog (7) and non-human primates (8). The clinical signs were severe, but the reversal of fentanyl with naloxone was performed soon enough and the pig recovered fully within an hour after naloxone application.

## Concluding Remarks

To the authors' knowledge, this is the first report of intoxication with an FTP after ingestion in a pig. In order to prevent FTP ingestion or chewing, the patch should not be attached to the same side of the body as, or close to, the surgical site. Inflammation at the surgical site may cause itching, and the pig may accidentally remove the patch by rubbing its body against the pen. The interscapular region may be advantageous over the lateral thoracic region as the FTP or other transdermal patch application site since it may be harder for a pig to remove the patch from the dorsal part of the body. Adhesive dressing over the FTP should be also considered, and continuous video surveillance of experimental pigs should always be employed. After the FTP is clinically not useful anymore (beyond 72 h), it should be removed and properly discharged to avoid intentional or unintentional misuse in animals and humans. In case of accidental intoxication, naloxone efficiently reverses the toxic effects of fentanyl if it is administered early enough.

## Data Availability Statement

The original contributions presented in the study are included in the article/supplementary materials, further inquiries can be directed to the corresponding author.

## Ethics Statement

The study was approved by the National Ethics Committee and National Veterinary Administration (approval number U34401-1/2017/4, approval date 17.03.2017) and conducted in accordance with ARRIVE (Animal Research: Reporting of in Vivo Experiments) guidelines. The pig was reared according to the European Council directive for minimum standards for the protection of pigs (2008/120/EC), and all procedures complied with the relevant Slovenian (Animal Protection Act UL RS, 43/2007) and European (Directive 2010/63/EU) guidelines.

## Author Contributions

JS, MB, UL, MC, and AS were involved in clinical management of the case. TK performed laboratory analyses. MC, MK, and AS designed and supervised the experiment. JS, TK, MK, and AS discussed the case and drafted the manuscript. All authors reviewed, edited, read, and approved the final manuscript.

## Conflict of Interest

The authors declare that the research was conducted in the absence of any commercial or financial relationships that could be construed as a potential conflict of interest.

## Abbreviations

FTP: fentanyl transdermal patch.
